# A SINE-VNTR-*Alu* in the LRIG2 Promoter Is Associated with Gene Expression at the Locus

**DOI:** 10.3390/ijms21228486

**Published:** 2020-11-11

**Authors:** Ashley Hall, Anni K. Moore, Dena G. Hernandez, Kimberley J. Billingsley, Vivien J. Bubb, John P. Quinn, NABEC (North American Brain Expression Consortium)

**Affiliations:** 1 Department of Pharmacology and Therapeutics, Institute of Systems, Molecular & Integrative Biology, University of Liverpool, Liverpool L69 7BE, UK; ashley.hall@liverpool.ac.uk (A.H.); jillbubb@liverpool.ac.uk (V.J.B.); 2 Laboratory of Neurogenetics, National Institute on Aging, National Institutes of Health, Bethesda, MD 20892, USA; anni.moore@nih.gov (A.K.M.); hernand@mail.nih.gov (D.G.H.); kimberley.billingsley@nih.gov (K.J.B.)

**Keywords:** SVA, polymorphism, retrotransposon, gene regulation, LRIG2, evolution, RIP, retrotransposon insertional polymorphism

## Abstract

The hominid SINE-VNTR-*Alu* (SVA) retrotransposons represent a repertoire of genomic variation which could have significant effects on genome function. A human-specific SVA in the promoter region of the gene leucine-rich repeats and immunoglobulin-like domains 2 (*LRIG2*), which we termed SVA_LRIG2, is a common retrotransposon insertion polymorphism (RIP), defined as an element which is polymorphic for its presence or absence in the genome. We hypothesised that this RIP might be associated with differential levels of expression of LRIG2. The RIP genotype of SVA_LRIG2 was determined in a subset of frontal cortex DNA samples from the North American Brain Expression Consortium (NABEC) cohort and was imputed for a larger set of that cohort. Utilising available frontal cortex total RNA-seq and CpG methylation data for this cohort, we observed that increased allele dosage of SVA_LRIG2 was non-significantly associated with a decrease in transcription from the region and significantly associated with increased methylation of the CpG probe nearest to SVA_LRIG2, i.e., SVA_LRIG2 is a significant methylation quantitative trait loci (mQTL) at the *LRIG2* locus. These data are consistent with SVA_LRIG2 being a transcriptional regulator, which in part may involve epigenetic modulation.

## 1. Introduction

Across many diseases, most disease-associated genetic variation identified by genome-wide association studies and gene candidate studies is located within non-coding DNA. A major constituent of non-coding DNA is sequence derived from retrotransposons, which constitute approximately 45% of the human genome [1]. Retrotransposon sequences propagate throughout the genome via a ‘copy-and-paste’ mechanism, first involving transcription into RNA followed by reverse transcription and insertion of a new copy at a distant genomic site. They are important contributors to genetic diversity via their ability to introduce novel splice sites, promoters and regulatory elements into a locus [2,3], thus facilitating the generation of novel species- and tissue-specific expression patterns in the germline.

**Figure 1:**
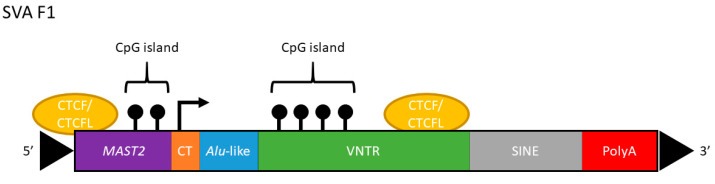
The canonical structure of the SVA F1 retrotransposon subclass, 5′ to 3′ displayed left to right. Purple: 5′ transduction of exon 1 of *MAST2*. Potential CCCTC-binding factor (CTCF) binding site and CpG island are displayed. Orange: The (CCCTCT)n hexamer repeat, which may be variable in copy number. Note, much of this sequence has been replaced by the *MAST2* transduction. Blue: The *Alu*-like region, which is composed of two antisense *Alu* regions separated by a small intervening sequence. Green: The VNTR region which may be one or two regions of 35–50 bp tandem repeats, and may reach GC content of 70%. A CpG island is pictured, with black lollipops signifying 5-methylcytosine residues. A potential binding site for CTCF is shown. Grey: The SINE region is derived from the 3′ LTR of the retroviral HERV-K10 element. Red: The canonical polyadenylated tail can act as a signal for transcription termination but may be bypassed by RNA polymerase, and is thought to be important for SVA retrotransposition. Black arrowheads indicate target site duplication regions resulting from insertion.

Non-long terminal repeat (non-LTR) retrotransposons are the only known contemporarily active retrotransposons in the human genome, of which SINE-VNTR-*Alu* (SVA) elements are the evolutionarily youngest [1]. Present only in hominids, there are approximately 3000 identified in the reference human genome (Hg38). Considered 5′ to 3′, SVAs are composed of a CT element (CCCTCTn repeat), an antisense *Alu*-like (another retrotransposon family) sequence, one or two variable number tandem repeat (VNTR) regions, a short interspersed nuclear element (SINE) region derived from the retroviral HERV-K10 element, and a poly-A tail [4] (Figure 1). SVAs do not transpose autonomously but are mobilised in *trans* by the transposition machinery of LINE-1, an autonomous class of non-LTR retrotransposon [5]. Full-length SVAs vary from 1 to 4 kb in size, and are classified A–F in order of evolutionary age based on their SINE region [4]. Additionally, an F1 class of SVA contains a 5′ transduction of exon 1 of the *MAST2* gene [6], such that much or all of the CT element is replaced (Figure 1). SVA subfamilies E, F and F1 are human specific, and with the addition of the D subfamily, are transpositionally active within the human genome [4]. It is estimated that one novel SVA insertion event occurs every 916 births, corresponding to 7 × 10^6^ distinct insertions worldwide [7]. A genomic site where a retroelement may be present or absent is known as a retrotransposon insertion polymorphism (RIP), and the average human is estimated to possess 56 SVA RIPs that differ from the reference genome [8]. Additionally, SVAs may be polymorphic with regards to the length (repeat copy number) of their CT element, VNTR and poly-A signal components, and may also contain single-nucleotide polymorphisms (SNPs). Accordingly, SVAs are considered the most polymorphic structural variants in the genome [8].

**Figure 2:**
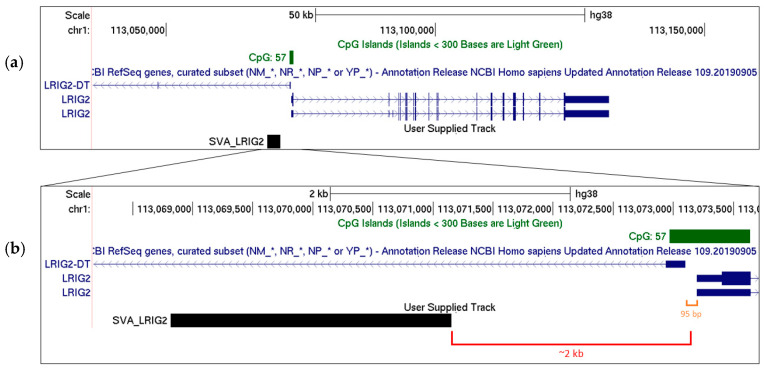
(**a**) The *LRIG2* locus as shown on UCSC genome browser hg38, displaying two validated LRIG2 isoforms and first exon of *LRIG2-DT* from the RefSeq genes curated subset, and the *LRIG2* promoter-associated CpG island. SVA_LRIG2 position is displayed. (**b**) Regions flanking SVA_LRIG2 are displayed, with distance to *LRIG2* locus transcriptional start sites highlighted in red. Distance between *LRIG2* and *LRIG2-DT* transcriptional start sites highlighted in orange.

On chromosome 1, there is a 2.4 kb long (full-length) SVA F1 positioned approximately 2 kb upstream from the transcriptional start site of leucine-rich repeats and immunoglobulin-like domains 2 (*LRIG2*) (Figure 2), a protein which is involved in epidermal growth factor signalling. The LRIG2 protein is ubiquitously expressed but deleterious mutations are associated with urofacial syndrome [9,10], a disorder characterised by improper nerve control of the face and bladder [11]. *LRIG2* shares its bidirectional promoter region with a non-coding divergent transcript, *LRIG2-DT*, whose expression could be similarly regulated [12,13,14] (Figure 2). The SVA proximal to this *LRIG2* locus (herein SVA_LRIG2) is a RIP, meaning that it transposed relatively evolutionarily recently, and may represent an insertion event involved in human-specific modulation of gene expression. Indeed, SVA RIPs and length polymorphisms may have profound effects on gene regulation and genomic architecture. Over 60% of SVAs in the reference genome are found in genes or within 10 kb of a gene despite this representing only approximately 1% of the human genome [15], suggesting preferential insertion into actively transcribed regions [4]. The GC content of an SVA is approximately 60% and may exceed 70% within the VNTR region, satisfying the definition of a CpG island [16]. SVAs have been shown to be methylated in somatic tissue, with evolutionarily older classes of SVA generally showing greater methylation [17]. DNA methylation is known to be capable of spreading laterally along DNA from CpG islands [18], and mouse B1 SINE retrotransposons have been shown to induce transcriptional repression by DNA methylation spreading [19]. An SVA insertion may therefore influence expression of nearby genes by altering local chromatin structure or recruiting factors depending on both the SVA’s primary sequence and epigenetic marks [20,21]. Indeed, the 5′ *MAST2* exon 1 transduction associated with SVA F1 elements is defined as a CpG island and has been demonstrated to influence transcription in human germline cells [22]. SVAs may also influence chromatin structure by recruiting CCCTC-binding factor (CTCF), a master regulator of 3D chromatin structure [23]. It has been demonstrated that CTCF can bind the SVA VNTR region in vitro, while the germline-expressed paralog CTCF like (CTCFL, also referred to as BORIS) can bind the VNTR region in vivo [21]. Interestingly, CTCFL has also been shown to bind immediately upstream of the SVA F1 subfamily [21].

Reporter gene studies have demonstrated that gene expression was differently modulated by different SVA components and the full-length SVA both in vitro and in vivo [15,24]. Indeed, genomic VNTRs have previously been shown to represent regions of stimulus responsive histone marks [25]. It should come as no surprise that insertion of SVAs into open reading frames is directly causative of at least 12 diseases due to transcriptional interference [26]. It is interesting to note that in X-linked Dystonia-Parkinsonism not only does a disease-specific SVA insertion appear causative of disease, but that the length of the CT hexamer repeat strongly correlates inversely with age of onset of the disease [27,28].

It is clear that SVAs represent non-coding DNA elements that have the potential to exert potent regulatory influences on nearby gene expression, and that RIPs or length polymorphisms between individuals may contribute to interpersonal differences in expression patterns. However, most work on SVAs thus far focus on their properties in vitro or in disease; there is currently a scarcity of data on the functional influence that SVAs may have in normal gene regulation in humans in situ. SVA_LRIG2 represents an opportunity to study the influence of an SVA insertion in situ as it is a RIP, meaning that DNA samples from the general populace represent a naturally occurring resource to address the influence of presence or absence of an SVA on genome structure or gene regulation. Indeed, the Database of RIPs in Humans (dbRIP) assigns an allele frequency of 0.422 to the presence of SVA_LRIG2 (dbRIP accession RIP3000013) [29], meaning that presence and absence of the SVA should be observed almost equally often. Furthermore, SVA_LRIG2 has a GC content of approximately 70% and contains 170 CpG dinucleotides, representing a potentially substantial CpG island. By contrast, the CpG island associated with the *LRIG2* promoter region, identified in the UCSC genome browser, is 669 bp long, approximately 65% GC content and contains 57 CpGs (Figure 2). We might postulate that this CpG island would be sensitive to methylation changes induced by the much larger one represented by SVA_LRIG2. To investigate the potential for this RIP to exert regulatory properties at the *LRIG2* locus we genotyped SVA_LRIG2 in DNA samples from the North American Brain Expression Consortium (NABEC), a cohort of neurologically normal frontal cortex samples from the Database of Genotypes and Phenotypes (dbGaP) for which molecular datasets including genome-wide genotyping, total RNA sequencing and CpG methylation were available.

## 2. Results

### 2.1. SVA_LRIG2 Genotypes Were PCR Validated or Imputed for 288 Individuals

**Figure 3:**
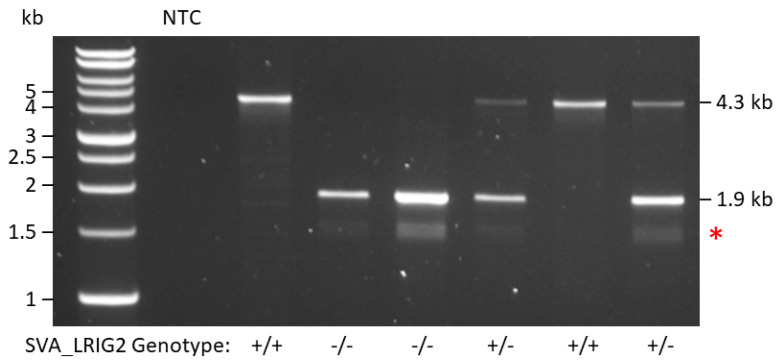
Representative image of NABEC DNA samples following PCR with primers targeting regions flanking SVA_LRIG2. A 1.9 kb PCR product corresponds to SVA_LRIG2 being absent from the locus and a 4.3 kb PCR product corresponds to SVA_LRIG2 presence. Sample genotypes are displayed along the bottom. NTC = Non-template control. Red asterisk indicates formation of non-specific PCR products of approximately 1.5 kb.

Although the NABEC cohort has undergone whole-genome sequencing (WGS), repetitive DNA elements such as retrotransposons are routinely filtered out due to their inherent difficulties in mapping back to the reference genome [30]. It was therefore necessary to genotype SVA_LRIG2 in the available samples from the NABEC cohort. Ninety-six brain DNA samples underwent PCR with forward and reverse primers that annealed 1.1 kb upstream and 0.8 kb downstream of genomic SVA_LRIG2, respectively. The inclusion of these flanking regions yielded a 1.9 kb PCR product when SVA_LRIG2 was absent from the locus and an approximately 4.3 kb PCR product when the SVA was present (Figure 3). This production of two distinctly sized PCR fragments allowed SVA RIP genotype (presence or absence) to be readily determined following agarose gel electrophoresis and visualisation. A representative gel is shown in Figure 3. Of the 96 samples genotyped, 14 had no copies of SVA_LRIG2 present at the locus (−/−), 43 had 1 copy present (+/−, i.e., the SVA was present on a single chromosome) and 39 had 2 copies present (+/+, i.e., the SVA was present on both chromosomes) (Table 1).

Although repetitive elements are not typically included in WGS processing and genotype calling, it is assumed that these variants may occur within haplotype blocks shared with variants through linkage disequilibrium. Therefore, the genotypes of these typically uncalled variants of interest may be inferred through ‘proxy’ (or ‘tagging’) SNPs that are in linkage disequilibrium [31]. In this way the genotype of SVA_LRIG2 can be imputed in NABEC individuals whose DNA was not available in our lab. We observed that SVA_LRIG2 exhibited length polymorphism with 4 VNTR alleles and developed proxy SNPs for each of them in order to fully capture the genetic diversity of SVA_LRIG2. This produced a total of 29 proxy SNPs, and the SNP with highest linkage disequilibrium r^2^ and D’ value for each VNTR allele was taken forward (see Materials and Methods). VNTR allele 4 was excluded after filtering for standard genotype missingness. The chosen proxy SNPs recapitulated the 96 PCR-validated genotypes with 97.4% accuracy (187 out of 192 alleles, data not shown). We then grouped these alleles into SVA_LRIG2 RIP genotypes: −/−, +/− and +/+. When added to the existing validated genotyping, this yielded 49 SVA_LRIG2 −/− genotypes, 154 SVA_LRIG2 +/− and 85 SVA_LRIG2 +/+, for a total of 288 individuals (Table 1).

### 2.2. Increased Allele Dosage of SVA_LRIG2 Is Non-Significantly Associated with Reduced Transcription from the LRIG2 Locus

**Figure 4:**
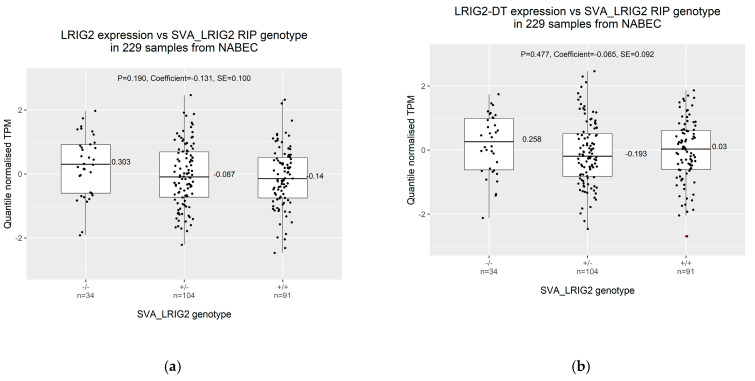
Frontal cortex total RNA-seq data for *LRIG2* (ENSG00000198799.12) and *LRIG2-DT* (ENSG00000238198.2) in 229 NABEC individuals grouped by SVA_LRIG2 RIP genotype. A total of 96 genotypes were PCR validated and 145 were imputed for a total of 241 genotypes. (**a**) *LRIG2* expression. (**b**) *LRIG2-DT* expression. RNA-seq data expressed as quantile normalised transcripts per kilobase million (TPM). Standard deviations from the mean of the normalised data are displayed on the y-axis. Linear regression analysis is shown, reporting p value of association analysis (P), model coefficient and standard error (SE). Outlier value is marked in red.

Having determined SVA_LRIG2 RIP genotypes experimentally or computationally, we assessed whether these genotypes correlated with transcription at the *LRIG2* locus. Of the 288 individuals in NABEC with validated or imputed SVA_LRIG2 genotypes, frontal cortex total RNA-seq data were available for 229. Expression values, expressed as quantile normalised transcripts per kilobase million (TPM), were extracted for *LRIG2* and *LRIG2-DT*. These expression values were stratified on the basis of SVA_LRIG2 RIP genotype, resulting in 34 individuals of the genotype SVA_LRIG2 −/−, 104 that were SVA_LRIG2 +/− and 91 that were SVA_LRIG2 +/+. Compared to those with no copies of SVA_LRIG2 (−/−), individuals with the RIP genotype SVA_LRIG2 +/− displayed a median expression of *LRIG2* that was 0.390 standard deviations lower and those with SVA_LRIG2 +/+ were 0.443 lower in the quantile normalised data (Figure 4a). Similarly, median expression of *LRIG2-DT* was 0.451 standard deviations lower in SVA_LRIG2 +/− individuals and 0.228 lower in SVA_LRIG2 +/+ individuals compared to those of genotype −/− (Figure 4b). A simple linear regression was used to assess whether SVA_LRIG2 was an expression quantitative trait loci in neurologically normal brain, i.e., whether increasing allele dosage of SVA_LRIG2 correlated with differential expression of *LRIG2* or *LRIG2-DT* in the NABEC RNA-seq data. The linear regression model included the known covariates age, gender, ethnicity, originating brain bank and RNA integrity number. Alpha significance level was set at 2.94E-3 using Bonferroni correction for multiple comparisons (see Section 4). Although we found that SVA_LRIG2 RIP genotype was not significantly associated with expression of *LRIG2* or *LRIG2-DT* (Figure 4, *p* = 0.190 and *p* = 0.477, respectively), our model indicated a negative trend between SVA_LRIG2 allele dosage and expression of both transcripts (Figure 4, negative coefficient values).

### 2.3. Increased Allele Dosage of SVA_LRIG2 Is Associated with Increased Methylation of the Nearest 450K Methylation Probe, cg23932873

**Figure 5:**
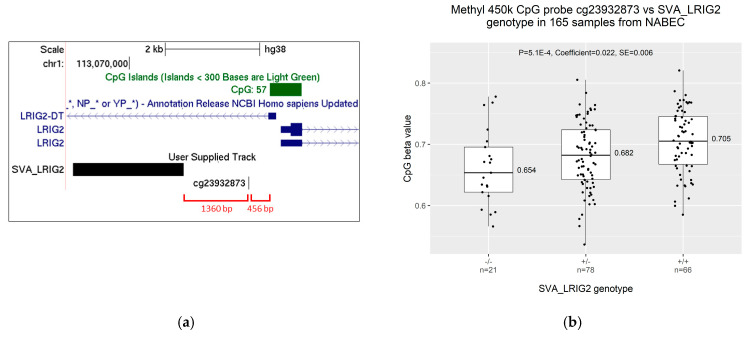
(**a**) The *LRIG2* promoter region as displayed on UCSC genome browser hg38, with two validated *LRIG2* isoforms and first exon of *LRIG2-DT* shown by the RefSeq genes curated subset. The position of SVA_LRIG2 is displayed in black. The position of cg23932873, the CpG probe closest to SVA_LRIG2, is shown in black and the *LRIG2* promoter-associated CpG island is shown in green. The distances in base pairs from cg23932873 to SVA_LRIG2 and the CpG island are shown in red. (**b**) Frontal cortex CpG 450K methylation data for probe cg23932873 in 184 NABEC individuals grouped by SVA_LRIG2 RIP genotype. A total of 96 genotypes were PCR validated and 88 were imputed for a total of 184 genotypes.

SVAs may simplistically be thought of as mobile CpG islands and DNA methylation is known to spread along adjacent DNA [16,18]. Therefore, we determined whether SVA_LRIG2 RIP genotypes might be associated with differences in methylation at the locus. Frontal cortex 450K CpG methylation data were available for 165 of the 288 individuals in NABEC with validated or imputed SVA_LRIG2 genotypes. Methylation data were again stratified on the basis of SVA_LRIG2 RIP genotype, resulting in 21 individuals of the genotype SVA_LRIG2 −/−, 78 that were SVA_LRIG2 +/− and 66 that were SVA_LRIG2 +/+. Publicly available ENCODE Methylation 450K Bead Array data list 15 CpG methylation probes in a 20 kb window around SVA_LRIG2, of which 13 are less than 3 kb away from the *LRIG2* promotor region (probes listed in Materials and Methods). As with the expression data, linear regression was used to determine whether SVA_LRIG2 was a methylation QTL (mQTL) for these 15 probes, i.e., whether increasing allele dosage of SVA_LRIG2 correlated with changes in CpG methylation. The linear regression model included the known covariates age, gender, ethnicity and originating brain bank. After Bonferroni correction for multiple comparisons (see Section 4), only the CpG dinucleotide probe nearest to SVA_LRIG2, cg23932873, was found to pass the adjusted alpha level of 2.94E-3 when methylation was correlated against SVA_LRIG2 genotype (Figure 5a). Compared to individuals with the genotype SVA_LRIG2 −/−, the median proportion of cg23932873 that was methylated was found to be 0.028 (2.8%) higher in those with the genotype +/− and 0.055 (5.1%) higher in those with SVA_LRIG2 genotype +/+ (Figure 5b). Our linear regression model provided a p value of 5.1E-4 and coefficient of 0.022, indicating that there is a significant association between SVA_LRIG2 allele dosage and increasing methylation of cg23932873. In other words, SVA_LRIG2 is a significant mQTL for a CpG dinucleotide at the *LRIG2* locus.

### 2.4. Increased Methylation of cg23932873 Is Weakly Correlated with Decreased Expression of LRIG2

**Figure 6:**
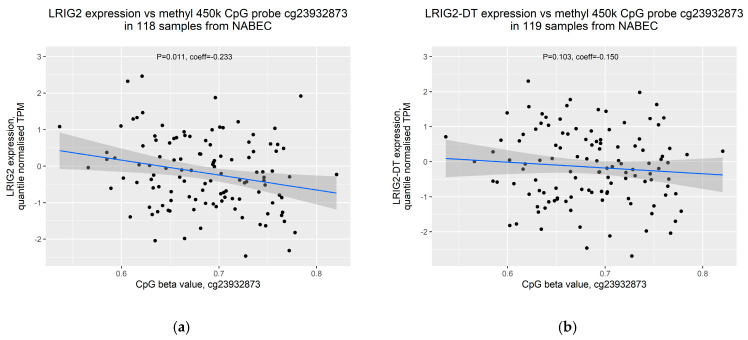
Expression of transcripts from the *LRIG2* locus versus CpG 450K methylation data for probe cg23932873 in frontal cortex of 129 NABEC individuals. (**a**) *LRIG2* (ENSG00000198799.12). (**b**) *LRIG2-DT* (ENSG00000238198.2). Blue line indicates trend line; dark grey zone indicates 95% confidence interval. Pearson correlation coefficient and corresponding p values displayed.

DNA methylation is known to generally repress gene expression through mechanisms including impairment of transcription factor binding and recruitment of proteins that confer repressive histone modifications [20]. Therefore, having observed that increased SVA_LRIG2 allele dosage is associated with decreased transcription from the *LRIG2* locus and increased methylation proximal to SVA_LRIG2, we hypothesised that the two may be correlated. Both RNA-seq data for *LRIG2* and CpG methylation data were available for 118 samples in the NABEC cohort after removal of outliers, while 119 samples were available for *LRIG2-DT* expression. CpG beta values for cg23932873 were plotted against normalised TPM values for *LRIG2* or *LRIG2-DT* and tested with Pearson correlation coefficient. We observed a weak yet significant inverse correlation between cg23932873 methylation and *LRIG2* expression (Figure 6a), and no correlation between cg23932873 methylation and *LRIG2-DT* expression (Figure 6b).

## 3. Discussion

Despite transposable elements making up nearly half of the human genome, they remain relatively unexplored as sources of important genomic variation [1], as their repetitive nature makes them difficult to map to the human genome. The evolutionarily youngest SVA retrotransposons may be particularly important for human health and evolution since they have introduced hominid-specific elements with regulatory potential to specific genomic loci, a process that is ongoing for SVA subclasses D–F1 [32]. It should be noted that SVA regulatory influences need not be dramatic to be biologically important in individuals of certain genetic backgrounds—for example, in the study of genetically complex disease, it is generally accepted that disease can result from the cumulative effect of many low-contribution variants [33]. Here we have investigated how presence or absence of an SVA retrotransposon 2 kb upstream of a divergent promoter region might influence regulation of transcription. SVA_LRIG2 is a relatively common RIP, meaning that the general populace represents a ready-made resource of the potential effects of SVA_LRIG2 presence vs absence at the *LRIG2* locus. The RIP genotype of SVA_LRIG2 was determined in the NABEC cohort using PCR and imputation and correlated with both gene expression and methylation at the nearby *LRIG2* and *LRIG2-DT* promoter region.

We observed a negative correlation between increased SVA_LRIG2 allele dosage and transcription of both *LRIG2* and the region’s divergent transcript *LRIG2-DT* in our linear regression models, but this did not achieve statistical significance. However, we note that this is not unexpected in a relatively small study of a heterogeneous population. Furthermore, the frontal cortex samples represent a tissue of heterogeneous cell types including neurons and glial cells, which may act to convolute expression and methylation patterns. Additionally, in this study, the SVA_LRIG2 genotype −/− is made up of a considerably fewer samples than genotypes +/− or +/+, and we speculate that expression differences may achieve statistical significance with an increased sample/cohort size. We postulate that this may be the case for *LRIG2* expression in particular, which displayed the clearest association between SVA_LRIG2 allele dosage and transcription. Despite falling short of statistical significance, these observations suggest that insertion of SVA_LRIG2 near to the *LRIG2* promoter region may be associated with decreased expression of transcripts from the locus, particularly *LRIG2*. That the two transcripts were similarly affected supports previous models in which divergent transcripts were co-regulated [12,13,14], and suggests a largely orientation-independent model for transcriptional modulation by SVA_LRIG2 at this locus.

When assessing the 15 CpG methylation probes in the region surrounding SVA_LRIG2, only the closest probe, cg23932873, was found to be a significant mQTL. Specifically, we found a strong positive association between SVA_LRIG2 allele dosage and levels of methylation of cg23932873 in the frontal cortex of individuals from the NABEC cohort. This indicates that SVA_LRIG2 may induce increased methylation of cg23932873, potentially via lateral spread of methylation from the GC- and CpG-rich SVA [16,18]. Furthermore, when expression from the *LRIG2* locus was compared directly to methylation of cg23932873 we observed a weak but significant inverse correlation, suggesting that increased methylation at cg23932873 induced by SVA_LRIG2 might contribute to the observed decrease in expression from the *LRIG2* locus. It has been demonstrated that methylated CpG dinucleotides are recognised by proteins associated with transcriptional repressor complexes [34], and that DNA methyltransferases can cooperate with enzymes that add methylation or remove acetylation at histones [35,36,37]. In this way, increased methylation at cg23932873 resulting from SVA_LRIG2 might contribute to formation of a transcriptionally repressive environment at the *LRIG2* locus, leading to the modest modulation of *LRIG2* and *LRIG2-DT* expression we have observed here.

We note that transcriptionally repressive effects of SVA_LRIG2 may also be mediated by other properties of SVA F1s that are not necessarily mutually exclusive to increasing local methylation. For example, binding of the chromatin structural regulator CTCF/CTCFL to SVA_LRIG2, either within the VNTR or upstream of the *MAST2* transduction [21], may form novel chromatin loops that preclude interactions between the *LRIG2*/*LRIG2-DT* promoter region and enhancer elements [23]. However, only expression and methylation datasets were available in the NABEC cohort, meaning that analysis of the influence of SVA_LRIG2 on 3D chromatin structure is currently beyond the scope of this study. We also note that *LRIG2* and *LRIG2-DT* may be differently affected, particularly at the post-transcriptional level, by SVA_LRIG2 owing to its genomic context; SVA_LRIG2 is 2 kb upstream of the promotor region of both transcripts, but is within the first intron of *LRIG2-DT*. It has been demonstrated that intronic SVAs are associated with intron retention in mRNA [27,38], which may trigger various nuclear degradation mechanisms and result in decreased overall transcript levels [39].

Taken together, these results suggest that recent SVA insertion events may have the potential to fine-tune the expression of nearby transcripts, and that one potential mechanism for this is via modulation of methylation at the promoter region. These in situ observations expand upon previous work demonstrating the in vitro regulatory effects of SVAs [15,24]. This is pertinent when we consider that over 60% of SVAs are found in genes or within 10 kb of a gene [15], and may therefore influence a wide variety of biological processes. Given that the data presented here were derived from the NABEC cohort, our findings are specifically applicable to neurologically normal fontal cortex. Our observations therefore complement previous hypotheses that SVAs may be important sources of genomic variation in the central nervous system. As hominid-specific elements, SVAs may be implicated in the development of higher cognitive function and ongoing human-specific genome evolution [32]. It is also conceivable that transposable elements, particularly SVAs, which are currently filtered out of short-read WGS data represent an under-appreciated source of variation that may be relevant to genetically complex processes. This may be especially true for very recent, human-specific insertion events and human-specific physiology and evolution. We note that short-read sequencing technology produces reads of up to 600 bases, with inherent difficulties in mapping repetitive elements back to the genome. By contrast, ‘long-read’ sequencing regularly generates reads of over 10 kb [40], meaning large or repetitive structural variants such as retrotransposons may be readily detected in WGS. Indeed, recent advances and decreasing costs of long-read sequencing technologies should soon make its implementation routine [41]. In light of the work presented here, we expect that improved detection of retrotransposons in WGS projects will inevitably lead to greater emphasis on their roles as important structural variants in the immediate future of human genomics research.

## 4. Materials and Methods

DNA samples from the NABEC cohort (dbGaP accession phs001300.v1.p1) underwent PCR with 0.02 U/µl KOD Hotstart polymerase (Merck 71086, Darmstadt, Germany) and 1.5 mM MgSO_4_, 0.2 mM dNTPs, 1 M betaine, and 0.3 µM of each primer. To amplify SVA_LRIG2 and its flanking regions, the forward primer used was 5′-TCCACGGACTTCCTAGAACG-3′ and the reverse primer used was 5′-CGAGATGGCGGCAGTACC-3′. Thermal cycling conditions were 95 °C for 2 min, followed by 35 cycles of 95 °C for 20 s, 58 °C for 10 s and 70 °C for 1 min 50 s. Samples then underwent agarose gel electrophoresis at 110 V for 90 min and were visualised on a BioDoc-It™ UV transilluminator (Analytik Jena: UVP, Upland, CA, US).

In order to accurately capture the full genetic diversity of SVA_LRIG2, proxy SNPs were generated using the ‘--show-tags’ option in PLINK v1.90 [42] for each of the 4 SVA_LRIG2 VNTR region length variants observed in the 96 NABEC individuals validated in our lab (genotyping data not shown). For each variant, the proxy SNP with the highest r^2^ and D’ values was selected: VNTR 1 proxy rs114767321, r^2^ = 1, D’ = 1; VNTR 2 proxy rs183751190, r^2^ = 1, D’ = 1; VNTR 3 proxy rs12744009, r^2^ = 0.894, D’ = 1. Proxy SNPs for VNTR variant 4 were excluded after filtering for genotype missingness (<0.1). The remaining proxy SNPs for SVA_LRIG2 variants were then grouped into RIP genotypes: −/−, +/− or +/+.

Generation of the NABEC frontal cortex total RNA-seq data and CpG methylation data has been previously described in detail by Gibbs et al. [43]. Data relating to individuals under the age of 15 were excluded to minimize developmental effects in the data. Expression data were extracted for LRIG2 and LRIG2-DT with Ensembl accession numbers ENSG00000198799.12 and ENSG00000238198.2, respectively. CpG data samples with sex mismatch were excluded. We noted that Illumina’s Infinium MethylationEPIC manifest file listed cg23932873 as containing the SNP rs61818193 with MAF = 0.5, which would be a potential confounder of this association analysis. However, rs61818193 does not have a frequency listed on dbSNP and was absent in pre-filtered WGS data in the NABEC cohort. Therefore, we concluded it was acceptable to proceed with use of this methylation probe as an mQTL readout.

The CpG probes analysed at the *LRIG2* locus were: cg13503476, cg23932873, cg22598841, cg16709384, cg26091510, cg04139429, cg23175215, cg17310611, cg24448849, cg15031996, cg10983720, cg14912723, cg21504385, cg09332974, and cg23961141.

Linear regression analyses were performed in RStudio Version 1.2.1335 (Boston, MA, USA) using the ‘lm’ and ‘summary’ functions, where test statistics follow a Student’s t distribution. When assessing effects of SVA_LRIG2 genotype in RNA-seq and CpG methylation data for NABEC individuals, a total of 17 tests were performed: 2 RNA transcripts (*LRIG2* and *LRIG2-DT*) and 15 CpG probes were examined. The 0.05 alpha significance level for these linear regression analyses was therefore adjusted using Bonferroni correction for multiple tests: 0.05/17 = 2.94E-3.

## Figures and Tables

**Table 1:** SVA_LRIG2 RIP genotype counts in NABEC samples.

SVA_LRIG2 Genotype	Validated Counts	Imputed Counts	Total Counts
−/−	14	35	49
+/−	43	111	154
+/+	39	46	85
Total	96	192	288

## Abbreviations

NABEC	North American Brain Expression Consortium
SVA	SINE-VNTR-*Alu*
RIP	Retrotransposon insertion polymorphism
LTR	Long terminal repeat
Hg38	Human genome assembly GRCh38
VNTR	Variable number tandem repeat
SINE	Short interspersed nuclear element
SNP	Single-nucleotide polymorphism
CTCF	CCCTC-binding factor
CTCFL	CTCF like
LRIG2	Leucine-rich repeats and immunoglobulin-like domains 2
dbRIP	Database of RIPs in humans
dbGaP	Database of Genotypes and Phenotypes
WGS	Whole-genome sequencing
TPM	Transcripts per kilobase million
mQTL	Methylation quantitative trait loci
SE	Standard error
